# Identification and characterization of two novel KCNH2 mutations contributing to long QT syndrome

**DOI:** 10.1371/journal.pone.0287206

**Published:** 2024-01-05

**Authors:** Anthony Owusu-Mensah, Jacqueline Treat, Joyce Bernardi, Ryan Pfeiffer, Robert Goodrow, Bright Tsevi, Victoria Lam, Michel Audette, Jonathan M. Cordeiro, Makarand Deo

**Affiliations:** 1 Department of Electrical and Computer Engineering, Old Dominion University, Norfolk, Virginia, United States of America; 2 Masonic Medical Research Laboratory, Utica, New York, United States of America; 3 Department of Engineering, Norfolk State University, Norfolk, Virginia, United States of America; 4 Department of Computational Modeling and Simulation Engineering, Old Dominion University, Norfolk, Virginia, United States of America; 5 ICON Laboratory Services Incorporation, Whitesboro, New York, United States of America; University of Tampere, FINLAND

## Abstract

We identified two different inherited mutations in *KCNH2* gene, or human ether-a-go-go related gene (hERG), which are linked to Long QT Syndrome. The first mutation was in a 1-day-old infant, whereas the second was in a 14-year-old girl. The two *KCNH2* mutations were transiently transfected into either human embryonic kidney (HEK) cells or human induced pluripotent stem-cell derived cardiomyocytes. We performed associated multiscale computer simulations to elucidate the arrhythmogenic potentials of the *KCNH2* mutations. Genetic screening of the first and second index patients revealed a heterozygous missense mutation in *KCNH2*, resulting in an amino acid change (P632L) in the outer loop of the channel and substitution at position 428 from serine to proline (S428P), respectively. Heterologous expression of P632L and S428P into HEK cells produced no hERG current compared to the wild type (WT). Moreover, the co-transfection of WT and P632L yielded no hERG current; however, the co-transfection of WT and S428P yielded partial hERG current. Action potentials were prolonged in a complete or partial blockade of hERG current from computer simulations which was more severe in Purkinje than ventricular myocytes. Three dimensional simulations revealed a higher susceptibility to reentry in the presence of hERG current blockade. Our experimental findings suggest that both P632L and S428P mutations may impair the *KCNH2* gene. The Purkinje cells exhibit a more severe phenotype than ventricular myocytes, and the hERG current blockade renders the ventricles an arrhythmogenic substrate from computer modeling.

## Introduction

Long QT syndrome (LQTS) is characterized by a QT interval >450 ms and is associated with an increased incidence of ventricular arrhythmias in individuals leading to sudden cardiac death (SCD). To date, mutations in 15 genes have been identified, but the manifestation of LQTS is highly variable; some individuals develop severe arrhythmias such as Torsade de Pointes (TdP), whereas other individuals remain asymptomatic for many years. It is well established that mutations in cardiac ion channels are responsible for a significant number of LQTS cases, with mutations in *KCNH2* or human ether-a-go-go related gene (hERG) comprising about 45% of LQTS [1].

Variations of phenotype expression can underlie the severity of the disease-causing mutation as well as the co-existence of other genetic variations. In some instances, a single mutation in *KCNH2* is responsible for LQTS. In other instances, multiple mutations in the same gene can result in the disease [1, 2]. The presence of single nucleotide polymorphisms (SNPs) can also affect the phenotypic manifestation of LQTS [3]. These SNPs are not disease-causing by themselves, but the presence of SNPs can increase the arrhythmia susceptibility in the presence of another mutation. For example, we have previously demonstrated that the SNP K897T (in *KCNH2*) can accentuate the effects of another mutation in *KCNH2* [4]. The use of human induced pluripotent stem cell–derived cardiomyocytes (hiPSC-CMs) for models of genetic diseases has attracted attention in the treatment and understanding of human genetic diseases. Specifically, hiPSC-CMs derived from a patient’s own fibroblasts or blood can be de-differentiated into the pluripotent stage and then directed into the cardiac lineage. These patient-specific cardiomyocytes can then be utilized to study the alterations on the cardiac action potential (AP) produced by the mutation(s). Moreover, the alterations on the cardiac AP can be used to devise therapeutic strategies. It is not always possible, however, to obtain a fibroblast sample from a patient, especially in infants and young children.

In this study, we present two novel mutations in *KCNH2* gene in two different individuals who both presented with arrhythmias and LQTS. The first patient was a 1-day old infant who presented with a QTc = 510 ms and genetic analysis revealed a mutation in *KCNH2* resulting in an amino acid change of proline to leucine at position 632 (P632L). The second patient was first reported by Viskin et al. [5] with a QTc = 542 ms and carried a different mutation resulting in an amino acid substitution at position 428 from serine to proline (S428P). Transient transfection of either the P632L or S428P mutation into Human embryonic kidney (HEK) cells resulted in no measurable hERG current. Interestingly, co-transfection of WT with P632L resulted in no current, whereas WT/S428P co-transfection yielded current that was approximately 50% reduced compared to WT alone. Transfection of either mutation into WT hiPSC myocytes resulted in an electrophysiological phenotype (prolongation of AP) consistent with LQTS.

We also present a computer modeling approach to investigate hERG current blockade in 1D and 3D anatomical models. The effects of hERG current block on AP morphology in hiPSC myocytes, ventricular myocytes (VM) and cardiac Purkinje cells (PC), as well as the implications for reentry initiation in ventricles, were investigated in depth.

## Materials and methods

### ECG analysis

QT intervals were measured and adjusted to the heart rate (QTc), according to Bazett’s formula [6]. The end of the T wave was defined as the intersection with the isoelectric line of a tangent drawn to the descending portion of the T wave.

### Genetic evaluation

After informed consent was obtained, blood was collected from both patients. Genomic DNA was extracted from peripheral blood leukocytes and from fresh and frozen tissue with a commercial kit (Puregene, Gentra Systems, Inc. Minneapolis, MN, USA). The genomic DNA was amplified by polymerase chain reaction (PCR) on GeneAmp**®** PCR System 9700 (Applied Biosystems, Foster City, CA, USA). All exons and intron borders of the *KCNQ1*, *KCNH2*, *SCN5A*, *KCNE2* and *KCNE2* genes were amplified and analyzed by direct sequencing. PCR products were purified with a commercial reagent (ExoSAP- IT, USB, Cleveland, OH, USA) and directly sequenced from both directions using ABI PRISM 3100 Automatic DNA sequencer (Applied Biosystems, Foster City, CA, USA). Electropherograms were visually examined for heterozygous peaks and compared with reference sequences for homozygous variations (GenBank accession number NM_000219) using the CodonCode Aligner Ver. 2.0.4 (CodonCode Corporation, Dedham, MA, USA).

### Mutagenesis

*KCNH2* cDNA (accession No. NM_000238) in a bicistronic vector encoding green fluorescent protein (GFP) (GFIrHERG) was a kind gift from Dr. Connie Bezzina. The P632L or S428P mutation was introduced using the QuikChange II XL Site-Directed Mutagenesis Kit (Stratagene, La Jolla, CA, USA). The mutated plasmid was sequenced to confirm the presence of the mutation as well as the absence of other substitutions introduced by the DNA polymerase.

### hiPSC-CM culture

This investigation was approved by the Institutional Stem Cell Research Oversight (SCRO) Committee. Human iPSC (WiCell, Madison, WI, USA), passage 18–25, were maintained on growth factor-reduced Matrigel (Corning Corp., Corning, NY, USA) coated plates in E8 medium (Gibco, Gaithersburg, MD) with E8 supplement (Gibco, Gaithersburg, MD, USA). Maintenance and differentiation of hiPSC cells was performed as previously described [7–11].

Cardiac differentiation was induced by 6 μM CHIR99021 (Sigma-Aldrich, St. Louis, MO, USA) in RPMI1640 (Life Technologies, Carlsbad, CA, USA) medium containing B27 (minus insulin) and 50 μg/ml L-ascorbic acid (cell culture tested powder; Sigma-Aldrich, St. Louis, MO, USA) as a basal medium. After 24 h, media was changed in the same basal medium with 6 μM CHIR (Sigma-Aldrich, St. Louis, MO, USA). The following day, cells were kept in RPMI1640/B27 (- insulin) for 24 h. Medium was then replaced with similar basal medium, with the addition of 5 μM IWP2 (MedChem Express, Monmouth Junction, NJ, USA) and 10 μM KY0211 (MedChem Express, Monmouth Junction, NJ, USA) for days 2–6 changing media every other day. Afterwards cells were kept in RPMI1640/B27 (+insulin) for days 8–10 followed by a purification medium, RPMI 1640 without glucose (Life Technologies, Carlsbad, CA, USA) +4mM sodium L-lactate (Sigma-Aldrich, St. Louis, MO, USA). Cells were kept in purification medium for days 12–14, then back to basal medium of RPMI/B27 (+insulin) supplemented with 20 ng/ml triiodothyronine (T3, Sigma-Aldrich, St. Louis, MO, USA), and 1 μM dexamethasone (Cayman Chemical Co, Ann Arbor, MI, USA) until day 30. Single cells were dissociated around day 25 with 0.05% trypsin, 1 mg/ml collagenase II (Worthington, Lakewood, NJ, USA) and plated onto Matrigel coated dishes. After 5 days of recovery, recordings were taken. All experiments were performed on hiPSC-CMs 21 days post-differentiation.

### Transient expression in HEK cells

Chinese hamster ovary (CHO-K1) cells (ThermoFisher Scientific, Waltham, MA, USA) were grown in GIBCO F12 nutrient mixture (GIBCO, Invitrogen, Carlsbad, CA, USA) in 35-mm culture dishes and placed in a 5% CO_2_ incubator at 37°C. The cells were transfected using FuGene6 (Roche Diagnostics, Indianapolis, IN, USA). To assess the influence of wild type (WT) on expression of the mutant channels, CHO-K1 cells were co-transfected with various combinations of WT and P632L or S428P *KCNH2*. Electrophysiological studies were performed 48 to 72 hours after transfection on cells expressing fluorescence.

### Transient expression in hiPSC-myocytes

hiPSC myocytes were maintained in RPMI B27+ media in 35-mm culture dishes and placed in a 5% CO2 incubator at 37°C. To assess the influence of the mutant channels on WT channels, hiPSC myocytes were co-transfected with 1 μg of P632L *KCNH2* using Lipofectamine 2000. For this transfection studies, we presume the mutant plasmid co-assembles with endogenous WT. Electrophysiological studies were performed 72 hours after transfection on cells expressing green fluorescence.

### Electrophysiology recordings

Voltage clamp recordings of K^+^ currents were made as previously described [12] using patch pipettes fabricated from glass capillaries (1.5 mm O.D., Fisher Scientific, Pittsburgh, PA, USA). The pipettes were pulled using a gravity puller (Narishige Co. Ltd, Tokyo, Japan) and filled with pipette solution of the following composition (mmol/L): 40 KCl, 90 K-aspartate, 1.0 MgCl_2_, 10 HEPES, 10 NaCl, 5 MgATP and 10 EGTA, pH 7.2 (KOH). The pipette resistance ranged from 2–4 MΩ when filled with the internal solution. The perfusion solution contained (mmol/L): 130 NaCl, 5 KCl, 1.8 CaCl_2_, 1. MgCl_2_, 2.8 Na acetate, 10 HEPES, pH 7.3 with NaOH. Current signals were recorded using a MultiClamp 700A amplifier (Molecular Devices, Foster City, CA, USA) and series resistance errors were reduced by about 60–70% with electronic compensation. All signals were acquired at 10–50 kHz (Digidata 1322, Molecular Devices, Foster City, CA, USA) and analyzed with a microcomputer running pClamp 9 software (Molecular Devices, Foster City, CA, USA). Recordings were made at room temperature (RT) for HEK cells and 37⁰ C for hiPSC experiments. The HEK cells were held at −80 mV and membrane currents were elicited by stepping the voltage to membrane potentials between −50 and +70 mV in +10 mV increments. Rectification properties of WT, homozygous and heterozygous substrates which gives the full I-V relation were studied by activating channels at +40 mV and measuring the instantaneous current upon return to membrane potentials between −120 and +20 mV. The peak tail current during the recovering pulse is proportional to the maximum number of channels available at each voltage [12]. APs were recorded from HEK cells using whole cell ruptured patch method whilst the APs from monolayers were recorded using high resistance microelectrodes.

### Ca^2+^ transients and confocal analysis

Confocal Ca^2+^ imaging experiments were performed with an Olympus Fluoview laser-scanning confocal microscope (Olympus Life Science, Center Valley, PA, USA) as previously described [13]. Fluo 4-AM (dissolved in 20% F-127 pluronic in dimethyl sulfoxide (DMSO), final concentration 15 μM, Molecular Probes, Eugene, OR, USA) was added to hiPSC-CMs and incubated for 20 min at RT. Fluo-4 loaded hiPSC-CMs were placed in a perfusion chamber and excited at 488 nm using an argon laser, and fluorescence emission was detected via a 520-nm band-pass filter and photomultiplier tube. Confocal images were acquired with Fluoview acquisition software program and spontaneous activity was recorded on personal computer for later analysis. Images acquired with Fluoview acquisition software were analyzed with ImageJ and Fluoview analysis software (Olympus Life Science, Center Valley, PA, USA).

### Statistical analysis

All data are presented as Mean ± Standard Error of the Mean. Statistical comparisons were made using ANOVA followed by a student–Newman–Kuels, or Student’s t-test, as appropriate. Significance was determined at *p* < 0.05.

### Computer modeling

To elucidate our understanding of the arrhythmogenic potentials of the mutations in the *KCNH2* gene, we conducted computer simulations using single-cell and 3D models of cardiac excitation and propagation in openCARP software [14], an in-silico cardiac electrophysiology simulator. The experimental electrophysiology data from P632L and S428P mutations was incorporated into a recently developed hiPSC-CM model [15]. The hiPSC-CM model replicated the experimental data on the two mutations by upregulating the maximum conductance of the L-type calcium channel (I_CaL_), the transient outward potassium current (I_to_), and the rapid delayed rectifier potassium current (I_Kr_) by 10%, 18%, and 150%, respectively. Additionally, the maximum conductance in the Na^+^/K^+^ pump (I_NaK_), the Na^+^/Ca^2+^ exchanger current, ultra-rapid delayed potassium current (I_Kur_), and acetylcholine-activated inward-rectifying potassium current (I_KACh_) were downregulated by 69%, 71%, 78%, and 36%, respectively. We further studied the effects of the hERG mutations in adult rabbit and human biophysical models to assess the severity of I_Kr_ block in VMs and PCs. Single cell simulations were performed using rabbit PC [16], rabbit VM [17], human PC [18] and human VM [19] ionic models. For all ionic models, the hERG channel blockade was simulated by scaling the maximum conductance of I_Kr_ from 0% (control) to 100% (Complete blockade). The control and I_Kr_ block models were paced at a basic cycle length (bcl) of 1000 ms for at least 30s to attain a steady state. The consequence of I_Kr_ block on AP duration at 90% repolarization (APD_90_) and morphology were studied in both the PCs and VM cell types.

Three-dimensional simulations were performed using a rabbit ventricular mesh integrated with a His-Purkinje system (Fig 7A) to simulate whole heart response. The rabbit ventricular mesh and the His-Purkinje system were described elsewhere [20, 21]. Membrane activity of the ventricles and His-Purkinje system was represented by Mahajan et al. [17] and Aslanidi et al. [16] ionic models, respectively. The *KCNH2* mutation effects were implemented in the model by completely blocking the I_Kr_ current in both ventricular and Purkinje models. Sinus activity was simulated by applying stimulus at the top nodes of the His bundle at bcl of 1000 ms for 7s. The extracellular potentials were extrapolated to approximate limb electrode positions for leads I, II, and III to derive pseudo-ECGs.

The High-Performance Computing (HPC) facilities of Old Dominion University were used for all the numerical simulations in this study. The 3D simulations were run on 40 computing nodes with 2GB of physical memory per node.

## Results

### Genotype/Phenotype

**Fig 1:**
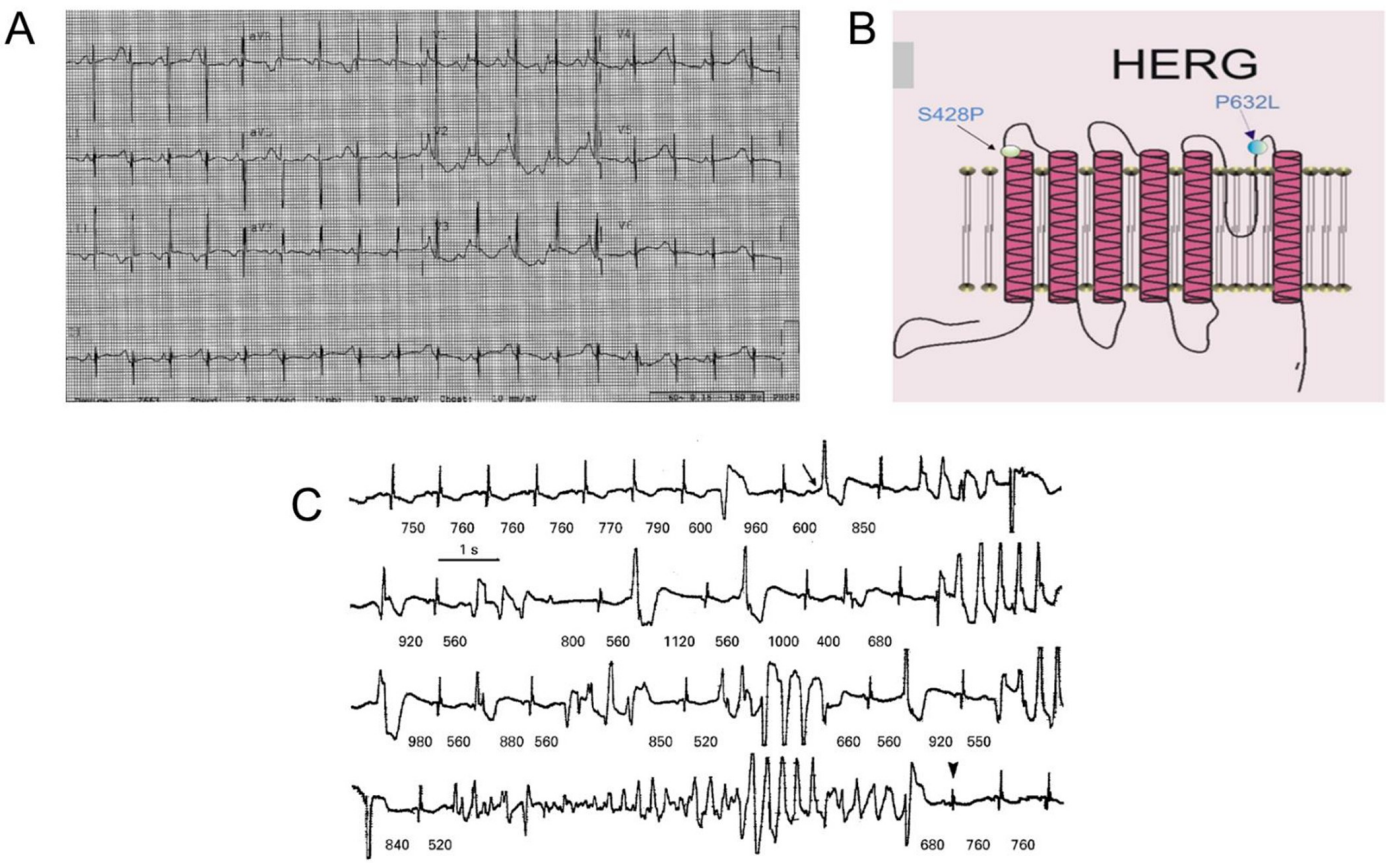
First index patient ECG, positions of *KCNH2* mutations and second index patient ECG. **A.** ECG of first index patient. ECG revealed prolonged QT and visible T-wave alternans. **B.** Cartoon of the *KCNH2* gene showing the location of P632L and S432P. **C.** Representative ECG of the second index patient. The R-R intervals is shown in milliseconds. Patient exhibited pause dependent torsade de pointes. Figure was taken from Viskin et al. [4].

The first index patient who carried the P632L mutation was a male newborn that exhibited runs of TdP and LQTS. Analysis of the ECG taken 1 day after birth revealed a prolonged QT interval (QTc = 510 ms measured in lead II) and visible T-wave alternans (TWA) (Fig 1A). The infant also showed right atrial enlargement and right ventricular hypertrophy. Implantable cardioverter-defibrillator (ICD) implantation was not performed due to his small size. The baby was monitored at the hospital until he gained enough weight to go ahead with the ICD implantation. Genetic analysis of the infant revealed a heterozygous nucleotide substitution of C to T at position 1895 of *KCNH2* predicting a substitution of proline for leucine at position 632 of hERG (Kv11.1) located in the P-loop/S6 region of the channel (Fig 1B).

The second index patient (S428P) was a 14-year-old girl when reported [5] had a QTc = 542 ms (Fig 1C). The patient exhibited multiple episodes of TdP following surgery for congenital biliary tract malformation. Pacemaker and beta-blockers were the therapeutic interventions used for the patient.

### Electrophysiological characterization of mutations

**Fig 2:**
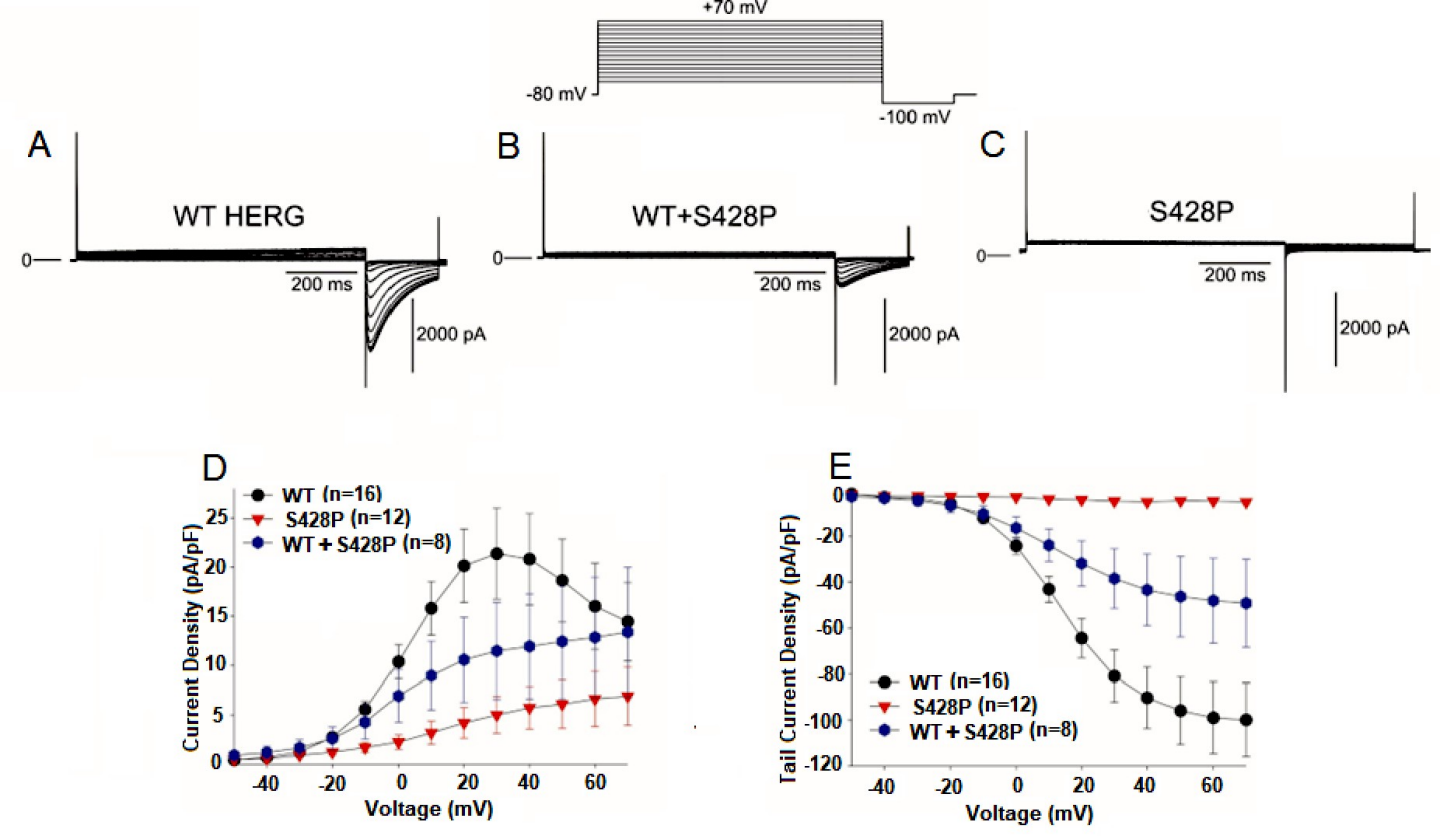
Activation and tail currents for WT, WT+S428P and S428P channels. **A.** Representative traces of WT HERG. **B.** Representative traces of WT+S428P. **C.** Representative traces of S428P channels. **D.** Current-voltage relationship for WT, S428P and WT+S428P channels measured at the end of the activating pulse. Activation currents were elicited by depolarizing pulses in +10 mV increments. **E.** Tail current–voltage relationship as a function of the activating step potential of WT, S428P and WT+S428P.The tail currents were generated by channels recovering from depolarization. Tail current amplitude reduced approximately by half in co-transfected channels when compared to WT**. WT: *n* = 16, WT+S428P: *n* = 12, S428P: *n* = 8**.

We then determined how the mutations altered the biophysical properties of hERG current and contributed to the clinical phenotype. We first examined the S428P mutation. WT and S428P Kv11.1 channels were expressed in HEK cells and patch clamp experiments were conducted. Recordings from WT cells activated rapidly during step depolarizations to positive potentials and showed tail current generated by channels recovering upon repolarization (Fig 2A–2C). S428P channels showed no activation (Fig 2D) nor tail currents (Fig 2E) compared to WT recordings. In Fig 2D, an activation current density of about 5pA/pF is recorded at potentials above 20 mV in cells transfected with S432P mutant channels. The recorded current magnitude, though significant in cardiomyocytes, is minuscule in transfected cells. In addition, the hallmarks of hERG current are not observed, namely inward rectification and rapid inactivation (S1D Fig in S1 File). Furthermore, the activation is not bell-shaped, and tail currents do not plateau at potentials above +10 mV [22]. The absence of current coupled with the absence of the hERG hallmarks led us to the conclusion that no activation was observed for the S428P mutation. Similarly, no current was observed by activating S428P mutant channels at +40 mV and measuring the instantaneous current upon return to membrane potentials between −120 and −40 mV (S1C and S1D Fig in S1 File).

**Fig 3:**
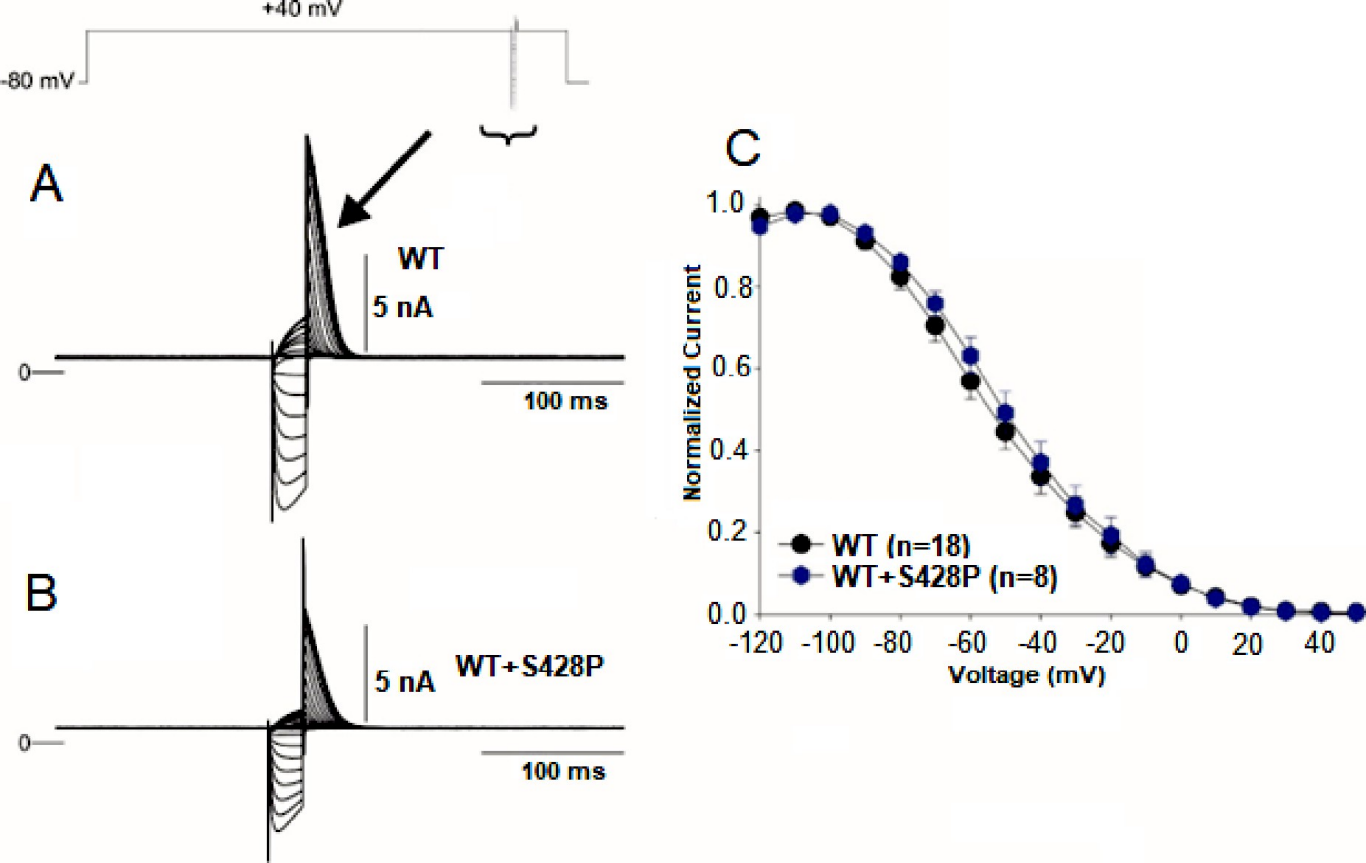
Channel availability recordings for WT and co-transfected WT&S428P channels using standard triple-pulse protocol. Channel availability recordings from a WT (**A)** and WT+S428P (**B**) using a standard triple-pulse protocol (top of figure). **C.** Plot of normalized current density versus voltage for WT and co-transfection of WT+S428P. WT and co-transfection of WT+S428P showed no significant difference in the mid inactivation values. **WT: *n* = 18, WT+S428P: *n* = 8**.

Interestingly, co-transfection of WT and S428P channels at a 1:1 molar ratio resulted in about a 50% reduction in current compared to WT recordings (Fig 2B, 2D and 2E). These results demonstrate that a heterozygous mixture of WT+S428P channels can form functional channels. Finally, we determined channel availability using a standard triple-pulse protocol (Fig 3). Mid-inactivation curve was obtained by fitting curve to a Boltzman function. Compared with WT, co-transfection of WT+S428P showed no significant difference in the mid-inactivation values. These results demonstrate that S428P channels yield non-functional channels but co-transfection of WT+S428P yields channels with reduced current.

**Fig 4:**
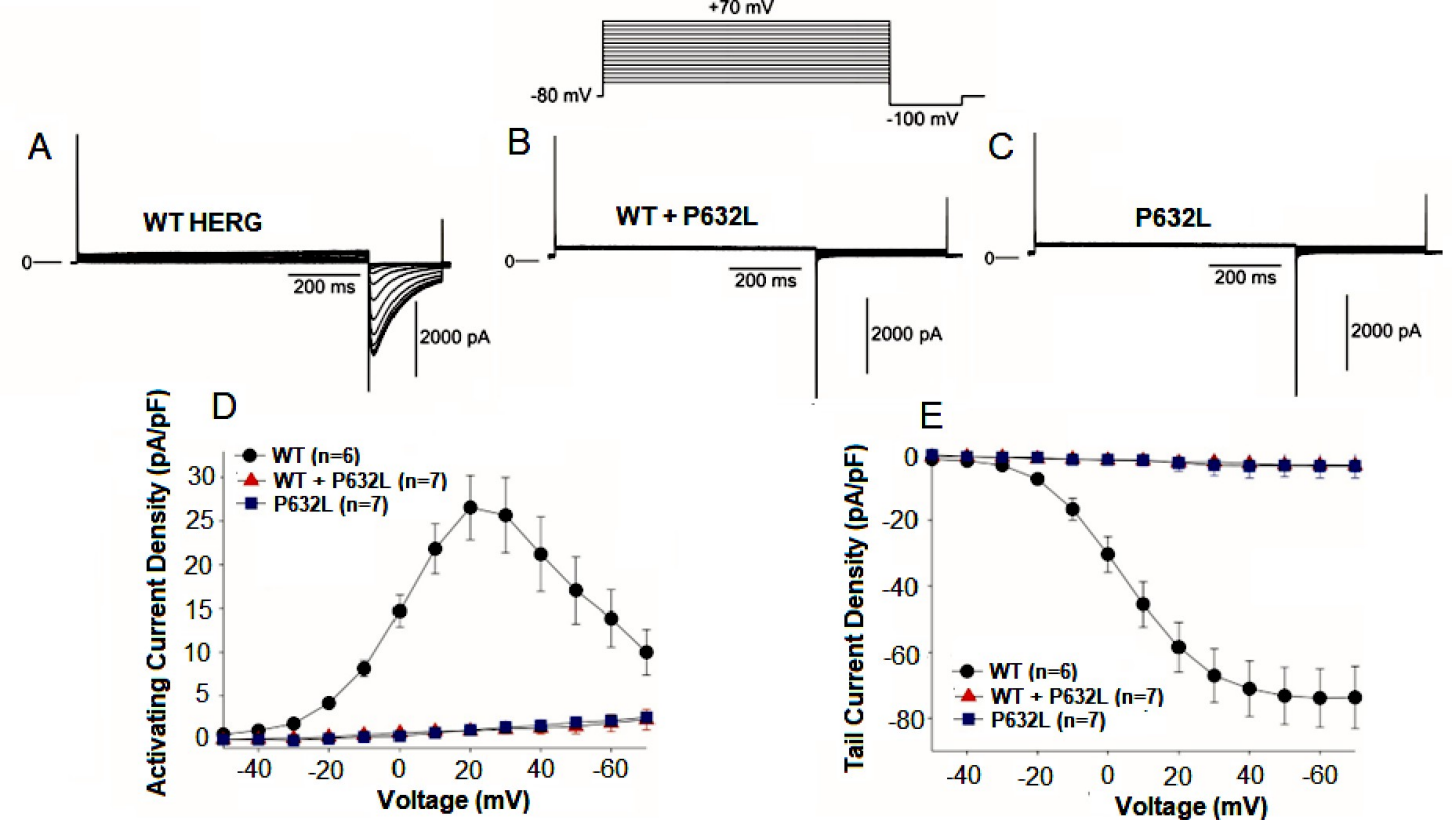
Activation and tail currents for WT, P632L and WT+ P632L channels. **A.** Representative traces of WT HERG. **B.** Representative traces of WT+P632L. **C.** Representative traces of P632L channels. **D.** Current-voltage relationship for WT, P632L and WT+ P632L channels measured at the end of the activating pulse. The cell was held at −80 mV and membrane currents were elicited by stepping the voltage to membrane potentials between −50 and +70 mV in +10 mV increments. **E.** Tail current–voltage relationship as a function of the activating step potential of WT, P632L and WT+ P632L.The tail currents were generated by channels recovering from depolarization. Tail current peak amplitude was zero for both the homozygous(P632L) and heterozygous (WT+P632L) substrates. **WT: *n =* 6, WT+ P632L: *n =* 7, P632L: *n* = 7**.

We next examined the P632L mutation identified in the 1-day old infant. WT or P632L Kv11.1 channels were expressed in HEK cells and patch clamp experiments were performed. Similar to results presented in Fig 2 for S428P channels, P632L channels yielded no activation nor tail currents compared to WT (Fig 4C–4E). In addition, no current was observed when a fully activated IV was applied to the cells (S2D and S2E Fig in S1 File). However, and in contrast to WT+S428P channels, co-transfection of WT+P632L channels at a 1:1 molar ratio resulted in no activation nor tail currents (Fig 4B, 4D and 4E). Fully activated IV also showed no measurable hERG currents for the heterozygous substrate (S2B and S2E Fig in S1 File).

Previous studies have reported that many nonfunctional channels can be rescued by a variety of methods, including incubation at RT or incubation with pharmacological agents such as I_Kr_ inhibitors to the culture media [23]. Our previous work [24] suggested trafficking impaired Na^+^ channel yielded larger current when rescued by RT incubation versus incubation with Na^+^ channel blockers. Based on our past results, we tested whether P632L channels could be rescued by culturing our transfected cells at RT before electrophysiological recordings. When the transfected HEK cells were incubated at RT (20°-22°C) for 48 hours before recordings were made, no effect on current size was observed (S2D Fig in S1 File).

Since mutations P632L and S428P resulted in non-functional channels when expressed in HEK cells, we next determined the functional effect of co-expressing either P632L or S428P with WT channels in hiPSC CMs. In these experiments, WT hiPSC CMs were transfected with mutant channels and the functional effect on APs were recorded. The results show that in hiPSC myocytes transfected with either P632L or S428P mutation, the myocytes exhibited prolonged APD_90_ compared to non-transfected (control) myocytes from the same differentiation batch (Table 1). Similarly, we recorded calcium transients in normal hiPSC-CMs and in those transfected with the P632L or S428P mutation from the same batch of hiPSC-CMs. Ca^2+^ transients from spontaneously beating hiPSC myocytes were recorded in cells loaded with fluo-4 AM. Compared to control recordings, Ca^2+^ transients from P632L or S428P transfected cells had a lower fluorescence intensity and exhibited a slower spontaneous rate (Table 2). In addition, irregular spontaneous rate and EAD-like activity in the fluorescence profile was noted in 30% of the cells transfected with P632L mutation (S3 Fig in S1 File).

**Table 1:** Electrophysiological parameters of WT, P632L and S428P -transfected hiPSC myocytes.

	APD50 (ms)	APD90 (ms)	Vmax (V/s)	Resting Membrane Potential (mV)	AP Amplitude (mV)
**Control (n = 14)**	133.1±18.6 ms	180.7±25.5 ms	24.5±1.1 V/s	-66.8±1.1 mV	95.6±1.1 mV
**P632L (n = 10)**	184.9±11.2 ms	247.0±18.2 ms*	26.5±1.7 V/s	-65.7±2.2 mV	96.6±3.0 mV
**S428P (n = 8)**	99.8±3.1ms	220.8±5.7 ms	29.3±1.8 V/s	-61.9±2.6 mV	104.7±3.2 mV

Values are given as mean ± Standard error of mean.

*p < 0.05 vs. control.

APD_50_ and APD_90_ are the action potential duration at 50% and 90% repolarization, respectively. Vmax = maximum upstroke velocity, AP = action potential. n = number of cells.

**Table 2:** Calcium transient parameters of WT, P632L and S428P -transfected hiPSC myocytes.

	Spontaneous Cycle Length	Number with Regular Spontaneous Cycle Length	% with Regular Spontaneous Cycle Length	(F-Fo)/F
**Control (n = 20)**	1705.6±54.8 ms	20/21	95.2%	5.23±0.33
**P632L (n = 16)**	1926.9±57.8 ms*	16/23	69.7%	3.03±0.38*
**S428P (n = 16)**	1711.8±145.8 ms*	16/20	80.0%	3.00±0.36*

Values are given as mean ± Standard error of mean.

*p < 0.05 vs. control. n = number of cells.

### Computer modeling

**Fig 5:**
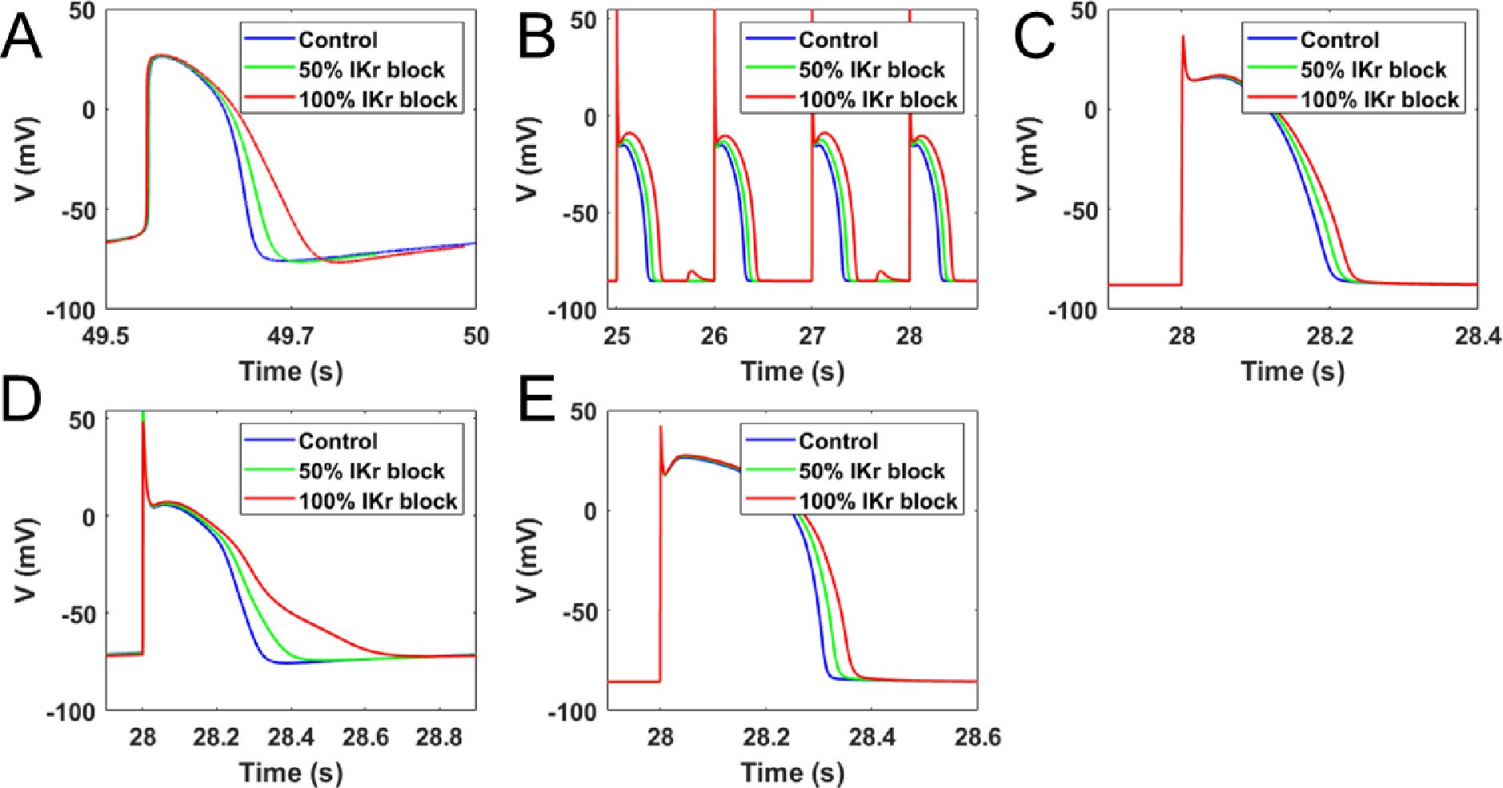
Evolution of action potential with time. **A.** Simulated AP of hiPSC-CM model for various percentages of I_Kr_ block (0%, 50% and 100%). Action potential duration prolonged with increasing I_Kr_ conductance block. **B.** Simulated AP of rabbit Purkinje cell (RPC) model for various percentages of I_Kr_ block (0%, 50% and 100%). Action potential duration prolonged with increasing I_Kr_ conductance block. 100% I_Kr_ blockade produced DAD. **C.** Simulated action potential of a rabbit ventricular myocyte (RVM) model for various percentages of I_Kr_ block (0%, 50%, and 100%). **D.** Simulated action potential of a human Purkinje cell (HPC) model for various percentages of I_Kr_ block (0%, 50%, and 100%). **E.** Simulated action potential of a human ventricular myocyte (HVM) model for various percentages of I_Kr_ block (0%, 50%, and 100%).

The effects of varying extents of I_Kr_ blockade were studied in a biophysical model of hiPSC-CMs. A complete blockade of I_Kr_ in the hiPSC myocyte model resulted in an approximately 39% prolongation in APD_90_ (Fig 5A) and approximately 2% reduction in frequency of spontaneous activity. We then studied the effects of these mutations in adult rabbit and human myocytes. Fig 5B and 5C show the simulated APs for rabbit PC (RPC) and rabbit VM (RVM) ionic models, respectively. I_Kr_ blockade increased APD_90_ in both models (14% and 42% for RVM and RPC, respectively for 100% I_Kr_ blockade). In addition, 100% I_Kr_ blockade in the RPC model produced delayed after depolarizations (DADs) and AP alternans which were absent in the RVM model. Fig 5D and 5E show the simulated APs for the human VM (HVM) and human PC (HPC) models. Blocking I_Kr_ entirely increased APD_90_ by 15% and 70%, respectively, in HVM and HPC. It is worth noting that, for a given I_Kr_ percentage block, change in APD_90_ is more pronounced in the human ionic models than rabbit models. In both human and rabbit models, I_Kr_ blockade was more severe in PCs than VMs. Table 3 lists the percent prolongation in APD_90_ for various extents of I_Kr_ block in VM and PC.

**Table 3:** APD_90_ and percentage prolongation for 0%, 50% and 100% I_Kr_ block in ventricular myocyte (VM) and Purkinje cell (PC) ionic models of human and rabbit.

	APD90 (ms)				
Ionic Models	Control	50% I_Kr_ block	100% I_Kr_ block	% Prolongation from control with 50% I_Kr_ block	% Prolongation from control with 100% I_Kr_ block
hiPSC-CM model	136	161	189	18	39
Rabbit VM (RVM)	211	225	241	7	14
Rabbit PC (RPC)	303	352	431	16	42
Human VM (HVM)	309	330	356	7	15
Human PC (HPC)	299	351	509	17	70

**Fig 6:**
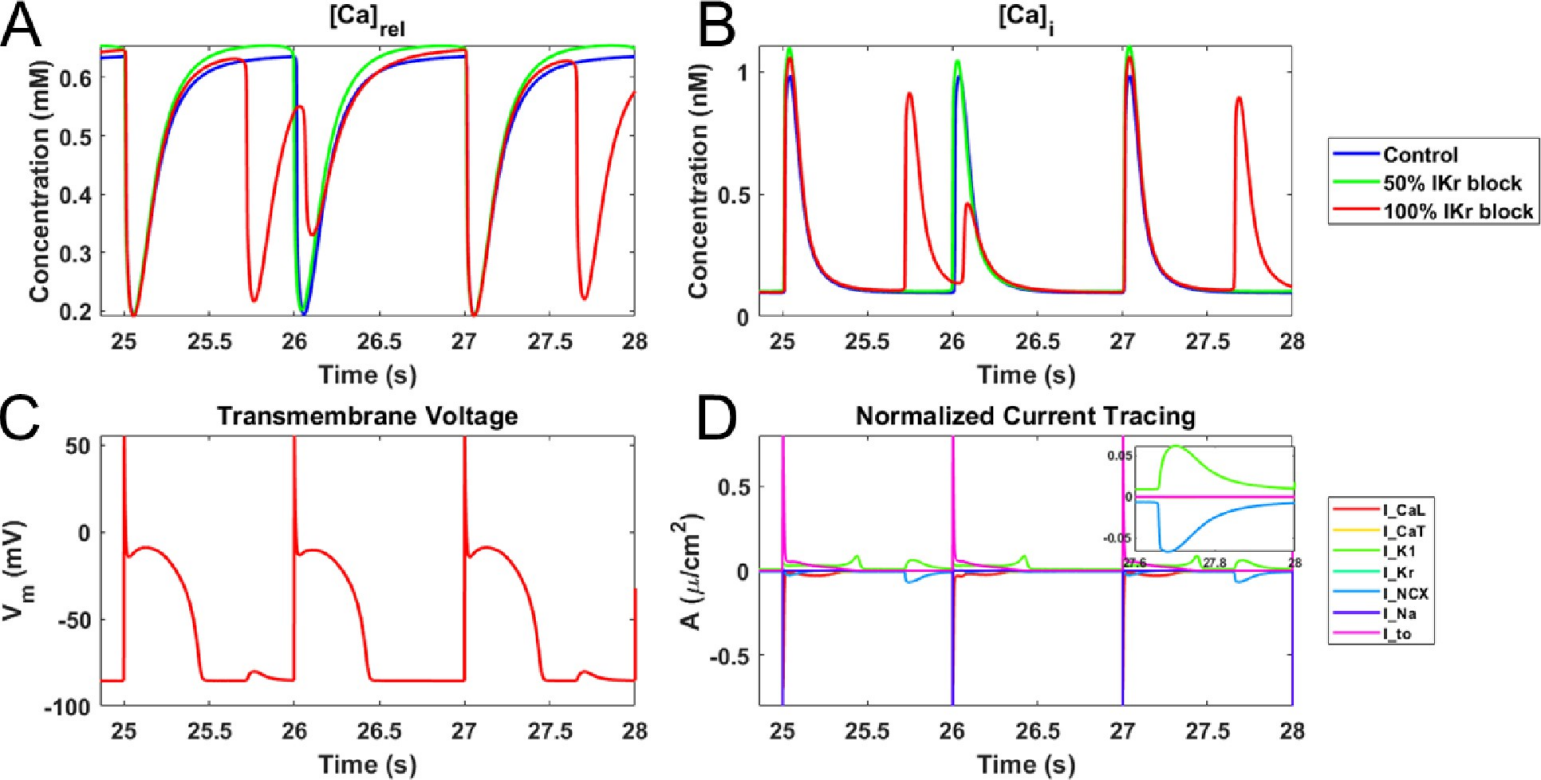
Delayed Afterdepolarization (DAD) activity window of the rabbit Purkinje cell model. **A.** Intracellular calcium concentration within the junctional SR of rabbit Purkinje cell (RPC) model during action potential (AP) for various percentages of I_Kr_ block (0%, 50%, and 100%). 100% I_Kr_ blockade produced DAD. **B.** Intracellular calcium in the cytosol of the rabbit Purkinje cell (RPC) model for various percentages of I_Kr_ block (0%, 50%, and 100%). **C.** Simulated AP of rabbit Purkinje cell (RPC) model for 100% I_Kr_ block. **D.** Current tracing of the various ionic currents that underlie the RPC model AP for 100% I_Kr_ blockade within a DAD activity window. Within a DAD activity window, the sodium-potassium exchanger current (INCX) and the inward rectifying potassium current (I_K1_) do not get to zero at the resting phase of AP.

We further investigated the underlying ionic mechanism during the occurrence of DADs in the RPC when I_Kr_ was completely blocked. Fig 6A shows that calcium is spontaneously released from the sarcoplasmic reticulum (SR) into the cytosol via ryanodine receptors (J_rel_) within a temporal window that causes the intracellular calcium to elevate rapidly (Fig 6B). The sodium-calcium exchanger (NCX) responds to the elevated levels of intracellular calcium by extruding the calcium ions in exchange of sodium ions, thus causing a net inward current and membrane depolarization leading to the formation of DADs (Fig 6D—inset).

**Fig 7:**
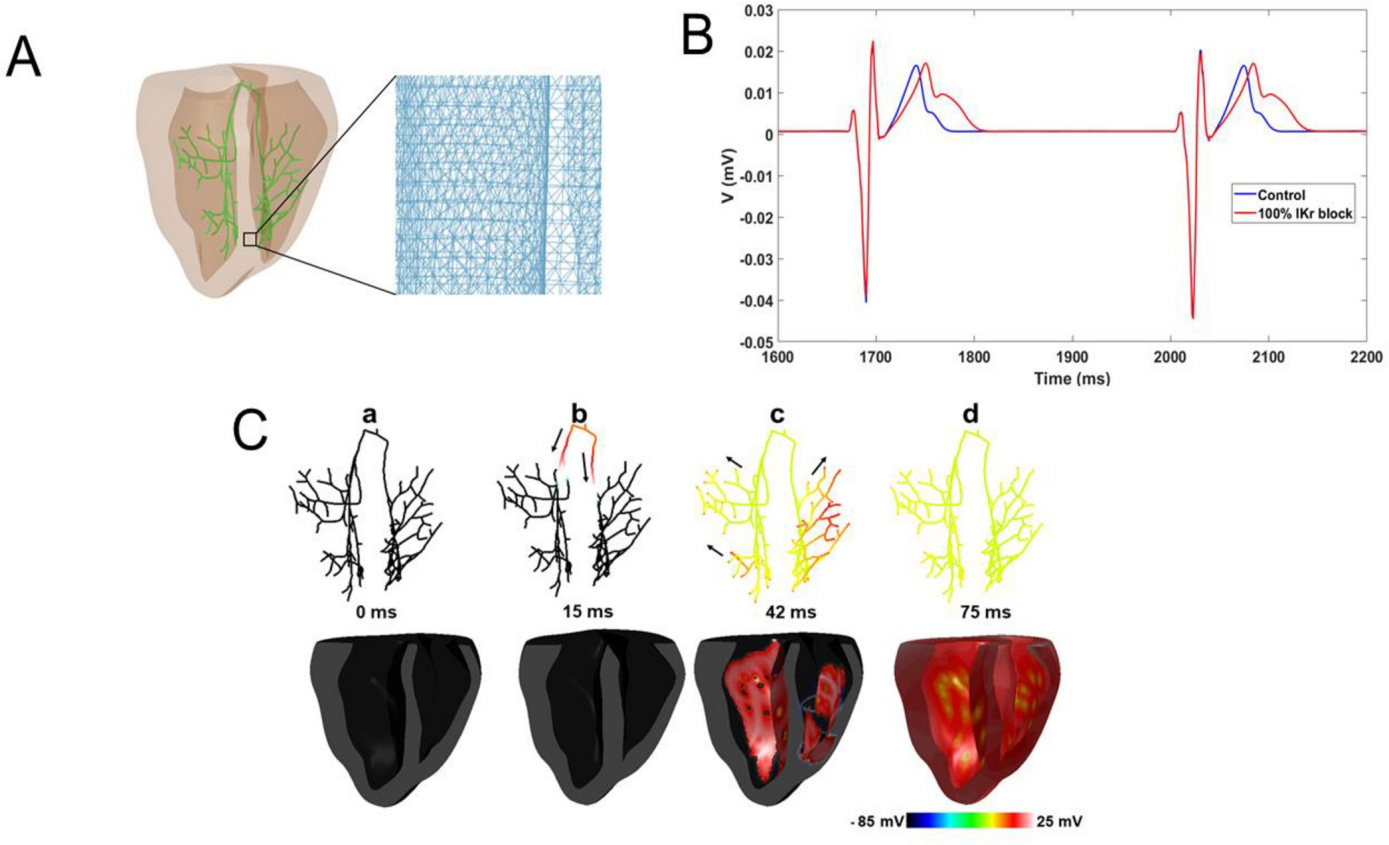
3D anatomical simulations. **A.** 3D computational mesh of rabbit ventricles integrated with a His-Purkinje system (HPS) (green color) used in our study [20, 21]. The inset shows the mesh discretization. **B.** Pseudo ECG recording for Control and 100% I_Kr_ block during sinus rhythm. **C.** Sequential snapshots of excitations (Snapshots were taken from the control model) during sinus rhythm in cross-sectional view of the ventricles (Lower) and the conduction through the HPS (Upper).

Fig 7A shows the biventricular model of rabbit anatomy with integrated Purkinje system network used in the 3D simulations. Fig 7B shows a representative pseudo-ECG trace during normal sinus rhythm in control and I_Kr_ block scenarios in the 3D anatomical model of rabbit ventricles. Complete I_Kr_ blockade prolonged the QT interval and increased the notch prominence in the T-wave. The QT duration measured was 104 ms and 138 ms for the control, and 100% I_Kr_ block, respectively (33% prolongation). Fig 7C shows a typical activation sequence in ventricles during sinus rhythm. The sinus beat is simulated by stimulating the top nodes of His bundle (Panel a in Fig 7C). The His excitation is propagated anterogradely through the bundle branches (Panel b) and the Purkinje-Myocardial junctions (Panel c), spreading to both ventricles simultaneously (Panel d).

**Fig 8:**
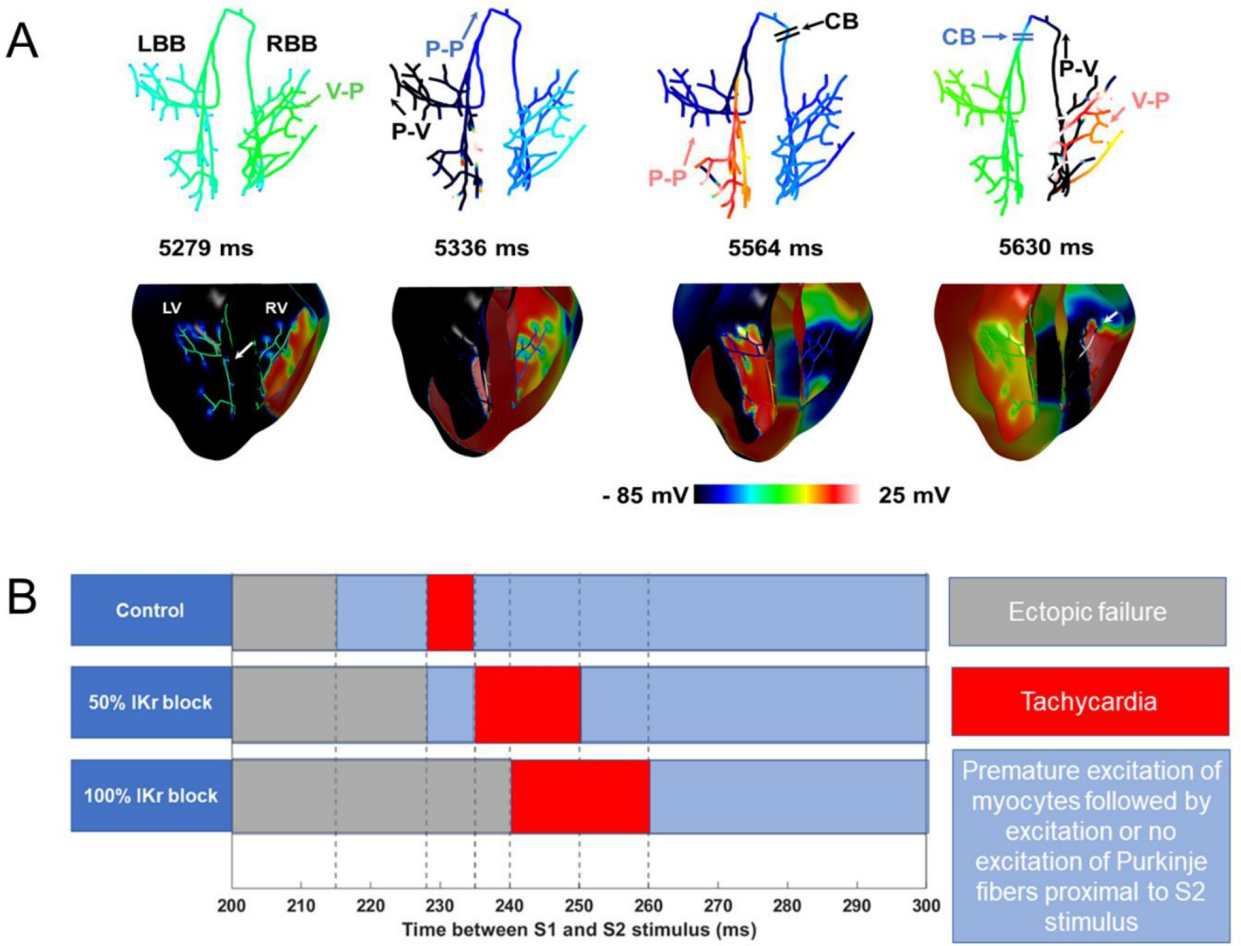
Reentry in 3D simulation. **A.** Reentry activations in the 3D ventricular model induced by an ectopic stimulus 252 ms post His-bundle activation. V-P: ventricular myocardium to Purkinje propagation; P-V: Purkinje to ventricular myocardium propagation; P-P: propagation within the Purkinje system; CB: conduction block. **B.** Conduction outcomes during reentry induction by S1-S2 window.

Fig 8 shows the simulated reentry mechanism when an ectopic stimulus was applied 252 ms after the last sinus beat in the 100% I_Kr_ block model and the window of vulnerability to reentry for the timing of the ectopic stimulus in the 3D simulations. At 5279 ms (Panel a in Fig 8A), the premature stimulus(S2) excited the myocytes in its vicinity. Excitation from the myocytes is propagated to the distal Purkinje fibers in the right bundle branch (RBB). Since the RBB was refractory, however, retrograde propagation through the RBB is blocked. Excitations reached the left ventricle (LV) via transeptal conduction. At 5336 ms (Panel b), due to the slowed conduction in the myocardium, activations reached the left bundle branch (LBB) now excitable, exciting the LBB and the LV. The RBB was now excitable afterwards, allowing retrograde propagation through the LBB to propagate anterogradely through it. At 5564 ms (Panel c), retrograde propagation though the LBB is impeded by the refractory RBB. At 5630 ms (Panel d), ventricular excitations from the RV are picked by distal Purkinje fibers in the RBB. The refractory LBB blocks retrograde propagation through the RBB.

Fig 8B shows the window of vulnerability to reentry due to S2 stimulus arriving at various intervals after the last His stimulus (S1). When the S2 stimulus occurred too early, it was blocked because most of the ventricles were refractory (grey region). If the S2 stimulation occurred too late, there was premature excitation of the entire ventricular tissue, and no reentry was observed (blue region). When the stimulus occurred while the ventricular tissue was partially excitable, complex interactions led to reentry and eventual tachycardia (red region). It is evident from Fig 8B that the blockade of I_Kr_ channels led to significant prolongation of the window of reentry that degenerates into tachycardia, thus highlighting the increased susceptibility to arrhythmia in presence of LQT2 conditions.

## Discussion

The results of our study show that the mutation P632L or S428P in *KCNH2* can potentially alter the function of hERG, resulting in various expressions of LQTS in patients. Transfection of WT channels into HEK cells yield hERG current. Both P632L and S428P yielded no measurable hERG current when transfected into HEK cells. Co-transfection of WT and S428P yielded hERG currents of reduced magnitude whereas WT and P632L resulted in no current, suggesting a dominant negative interaction. These observations may explain why the P632L mutation resulted in a severe phenotype in patients. The index patient developed arrhythmias, TWA and arrhythmias shortly after birth, strongly suggesting repolarization abnormalities; analysis of the ECG confirmed a QTc = 510ms. Subsequent genetic analysis revealed a mutation in *KCNH2* leading to an amino acid change at position 632 (P632L). Functional studies performed using cells expressing P632L hERG channels resulted in almost a complete loss of function, both the homozygous and heterozygous state. This profound loss may explain the severity and early onset of cardiac distress in the index patient.

The spontaneous release of calcium from the SR, which activates Ca-sensitive currents such as sodium-calcium exchanger current (I_NCX_), has primarily been linked to the mechanism of DADs [25]. The activity of I_NCX_ is reversed when intracellular Na^+^ increases, resulting in the efflux of Na and influx of Ca [26]. Similarly, cytosolic Ca^2+^ overload activates forward I_NCX_ leading to membrane depolarization and the eventual formation of DADs. Our single cell simulations revealed the spontaneous release of calcium from the junctional SR into the cytosol in the presence of complete I_Kr_ blockage leading to DADs in the rabbit PC model which was absent in the rabbit VM model. Many studies have reported that I_Ks_ protect the heart from excessive action potential prolongation induced by I_Kr_ inhibition [27]. As to why DADs were observed in the rabbit PC and not in the human PC model, when both models are paced at 1Hz frequency, the ratio of the peak I_Kr_ to I_Ks_ current density during action potential plateau is approximately 4 and 2, respectively, in the rabbit and human PC control models (S5A and S5B Fig in S1 File). Moreover, S5C and S5D Fig in S1 File provide evidence that when I_Kr_ was entirely blocked, the peak density current of I_Ks_ increased to compensate for the I_Kr_ loss. The peak I_Ks_ current after I_Kr_ inhibition in the human PC model is almost the same as that of the human control model’s I_Kr_ peak, whereas, in the rabbit PC model, the peak I_Ks_ current after I_Kr_ block is about 1/3 of the peak I_Kr_ current in the rabbit control model. These observations from S5 Fig in S1 File indicate that the I_Kr_ current is a significant repolarizing current in the rabbit PC model whose loss is not entirely compensated by I_Ks_ current, leading to the formation of the DADs. In contrast, in the human PC model, the I_Ks_ current density is relatively high to compensate for the loss of the I_Kr_ current.

Variations in the beat-to-beat AP and/or calcium transient provide a measure of TWA at the cellular level [28]. AP alternans at the cellular level is of several magnitudes higher than the corresponding magnitude in TWA on an ECG, which explains how even microvolt-level TWA observed on an ECG may be physiologically and clinically crucial [29].

Novel mutations in the *KCNH2* gene that were studied in [30–32] and de novo mutation in *KCNH2* [33] reported that the index patient experienced prolonged corrected QT and syncope. We only observed the prolongation in QT interval in our 3D simulation when I_Kr_ was blocked entirely, with no apparent beat-to-beat variability in the obtained pseudo-ECG T-wave. We surmise that this observation may be due to the obvious distinction in human and rabbit electrophysiology. Additionally, we prescribed a uniform ventricular AP across the ventricular mesh in the 3D simulation which abolished transmural-ventricular repolarization dispersion. The spatiotemporal dispersion of myocardial repolarization is certainly enhanced by alternans, which is the electrophysiological basis for TWA [28].

The pathogenic role of Purkinje fibers in ventricular arrhythmias has extensively been discussed [34, 35]. Propagation of subthreshold DADs can cause unidirectional conduction block in the presence of reduced excitability (reduction in Na conductance) that results in a vulnerable substrate for reentry [25]. From our single cell simulation of the RPC model, the maximum diastolic potential and DAD amplitude was -85.49 mV and -80.13 mV, respectively. In an attempt to simulate reentry in our 3D model, we reduced the Na conductance in both RPC and RVM ionic models by various percentages in control and I_Kr_ block models but were unsuccessful. The propagation of DADs generated in the Purkinje cells to the myocardium depends on the electrotonic interaction at the Purkinje-myocardial junctions. In our 3D simulations, it was observed that due to charging of the postjunctional membrane and the electronic loading of the coupled myocytes at the Purkinje Myocardial Junctions, DADs in the Purkinje cells were suppressed which prevented their propagation.

We had investigated the susceptibility to arrhythmia due to loss of I_Kr_ functionality in silico by an ectopic stimulus emanating from the right ventricle, utilizing a rabbit ventricular mesh and 1D branching network of the His-Purkinje system [21]. The timing of the ectopic stimulus to initiate sustained reentry as well as the window of susceptibility (Fig 8B, red region) to reentry increased with increasing I_Kr_ conductance block. The prolonged timing for reentry initiation can be attributed to the loss of I_Kr_ current, which prolongs the AP duration and dispersion in both VMs and PCs. Once the earliest time for the initiation of the stimulus is established, subsequent timings for the ectopic stimulation to initiate reentry follows, which reduce the blue region where an ectopic stimulus leads to premature excitation of the myocytes in its vicinity followed by excitation, or no excitation of the terminal PCs coupled to the myocytes.

hiPSC-CMs though nascent, have played a major role in advancing understanding of the pathophysiology of arrhythmogenesis. Whereas the *KCNH2* gene in transfected cells is studied in isolation, the *KCNH2* gene in native myocytes is influenced by other signaling pathways which can modulate its function. This indicates that expression systems may not necessarily reflect what is occurring in cardiac myocytes. The study herein did not categorize hiPSC-CMs as Ma et al. [36] did at different levels of repolarization. hiPSC-CMs categorization into ventricular, atria, or nodal-like cells was not performed in our study as the emphasis of our work was to characterize the electrophysiological effects of the two mutations in cardiomyocytes, irrespective of the cell type. As we did not include retinoic acid in the differentiation process, we presume that most cells are of the ventricular phenotype. In conclusion, the findings of this study suggest that mutations in *KCNH2* can lead to alterations in *KCNH2* function and the development of LQTS. Thus, screening for mutations in *KCNH2* can help in the effective diagnosis and management of LQTS.

## Supporting information

S1 File This file contains supplementary figures. These are the fully activated IV curve of S428P (**S1 Fig**), and P632L channels (**S2 Fig**), Calcium transients in normal hiPSC-CMs and hiPSC-CMs cells transfected with the P632L mutation (**S3 Fig**), response of rabbit Purkinje model to elevated level of sodium-calcium exchanger current following IKr blockade (**S4 Fig**), and IKs response following IKr blockade at 1Hz pacing (**S5 Fig**).
(DOCX)
