# Supplementary material for: Identification and characterization of two novel KCNH2 mutations contributing to long QT syndrome
Source: PLoS One. 2024 Jan 5;19(1):e0287206. doi: 10.1371/journal.pone.0287206 (PMC10769013; doi:10.1371/journal.pone.0287206)
Supplement: S1 File — These are the fully activated IV curve of S428P (S1 Fig), and P632L channels (S2 Fig), Calcium transients in normal hiPSC-CMs and hiPSC-CMs cells transfected with the P632L mutation (S3 Fig), response of rabbit Purkinje model to elevated level of sodium-calcium exchanger current following IKr blockade (S4 Fig), and IKs response following IKr blockade at 1Hz pacing (S5 Fig). (DOCX) [file pone.0287206.s001.docx]

**ONLINE SUPPLEMENTARY MATERIALS**

**Title: Identification and Characterization of Two Novel *KCNH2* Mutations Contributing to Long QT Syndrome**

**Authors:** Anthony Owusu-Mensah, Jacqueline A Treat, Joyce Bernardi, Ryan Pfeiffer, Robert J Goodrow^2^, Bright Tsevi, Victoria Lam, Michel Audette, Jonathan M. Cordeiro, Makarand Deo


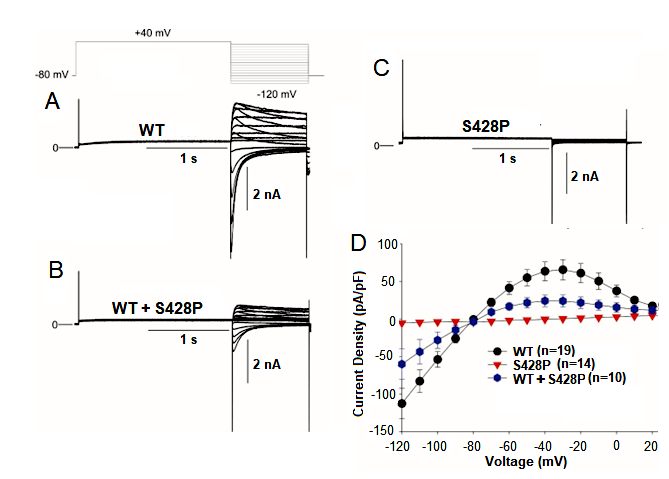


**Fig S1. Rectification properties of WT, S428P and WT+S428P channels.**

1. Representative traces of WT HERG fully activated currents. **B**. Representative traces of WT + S428P fully activated currents. **C.** Representative traces of S428P fully activated currents **D**. Peak tail current amplitude as a function of the test potential for WT, S428P and WT+S428P. WT and WT+S428P peak tail currents exhibited strong inward rectification characterized by a region of negative slope conductance for membrane potentials above −40 mV. **WT: *n* = 19, WT+S428P: *n* = 14, S428P: *n* = 10.**


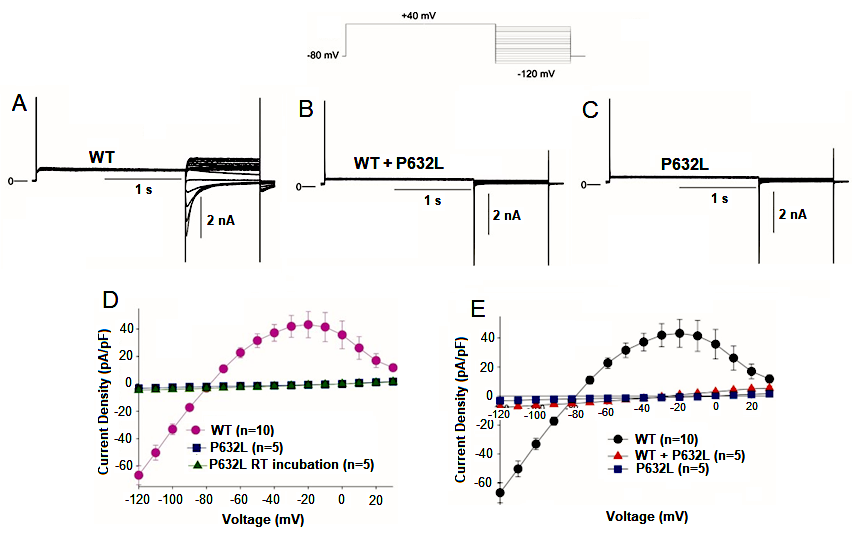


**Fig S2. Rectification properties of WT, WT+P632L, P632L and P632L incubated at room temperature (RT).**

1. Representative traces of WT HERG fully activated currents. **B.** Representative traces of WT+P632L fully activated currents. **C.** Representative traces of P632L fully activated currents. **D.** Peak tail current amplitude as a function of the test potential for WT, P632L and P632L incubated at RT. WT peak tail currents exhibited strong inward rectification characterized by a region of negative slope conductance for membrane potentials above −40 mV. RT incubation could not rescue mutant channels. **E.** Peak tail current amplitude as a function of the test potential for WT, WT+P632L and P632L. Both homozygous and heterozygous substrates showed no measurable current. **WT: *n* =10, WT+P632L: *n*=5, P632L: *n* = 5, P632L RT incubation: *n* = 5.**


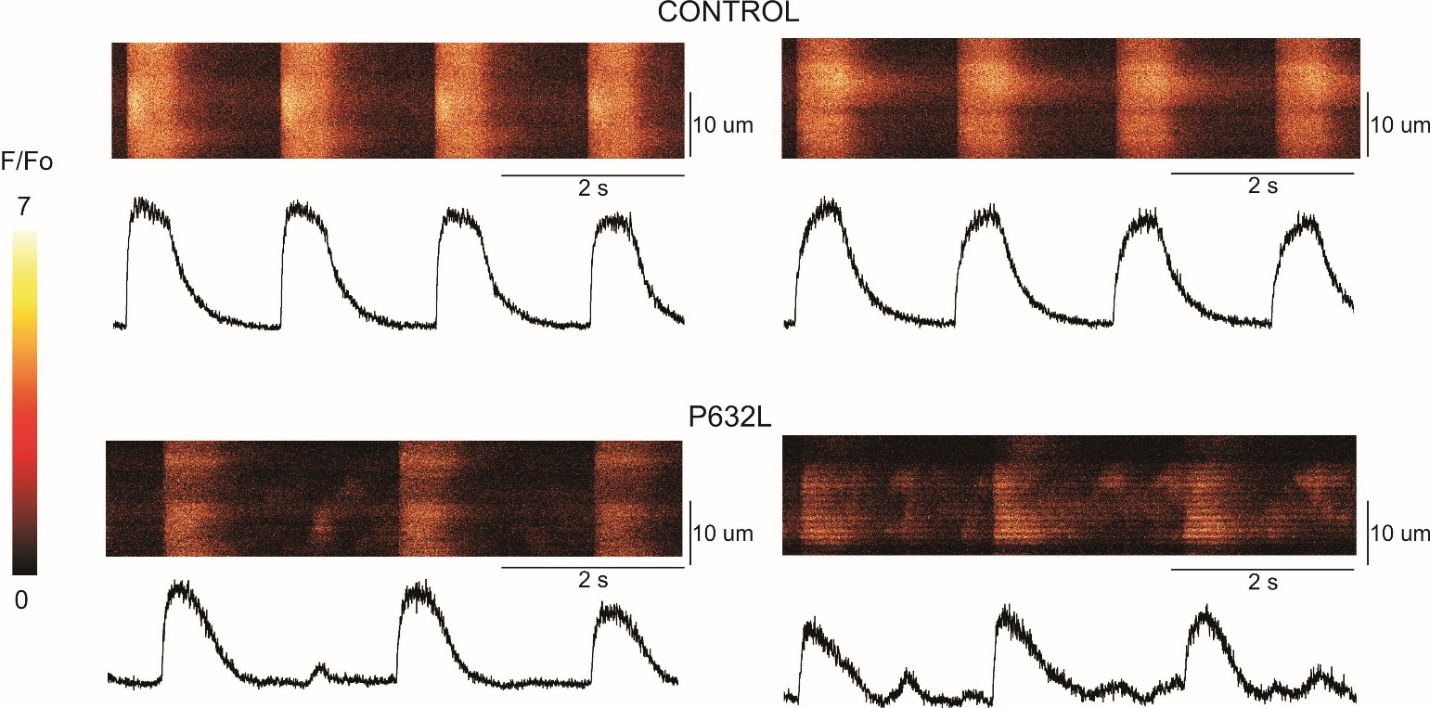


**Fig S3. Calcium transients in normal hiPSC-CMs and in hiPSC-CMs cells transfected with the P632L mutation.**

1. Calcium transients in normal hiPSC-CMs**. B.** Calcium transients in hiPSC-CMs transfected with the P632L mutation. Ca^2+^ transients from P632L transfected cells had a lower fluorescence intensity and exhibited a slower spontaneous rate. In addition, irregular spontaneous rate and EAD-like activity in the fluorescence profile was note in 30% of the transfected myocytes.

Within a temporal window, with IKr blocked entirely, the inward rectifying potassium current (IK1) and the sodium-calcium exchanger current (INCX) do not get to zero at the resting phase (around -80mV) of action potential in the rabbit Purkinje action potential model. The net current is an inward current that leads to the formation of DADs. To test whether the formation of DADs when IKr was blocked entirely resulted from the INCX, we raised the INCX current density to 2 folds. We paced the model at 2.5 Hz for 30 seconds (black arrow) and studied the evolution of the membrane potential with time after the stimulus had been taken off. With INCX raised to 2 fold and the stimulus is taken off, this led to a triggered activity (blue trace with yellow arrow). In contrast, no triggered activity was observed with the inward rectifying potassium current raised to 2 folds and IKr completely blocked.


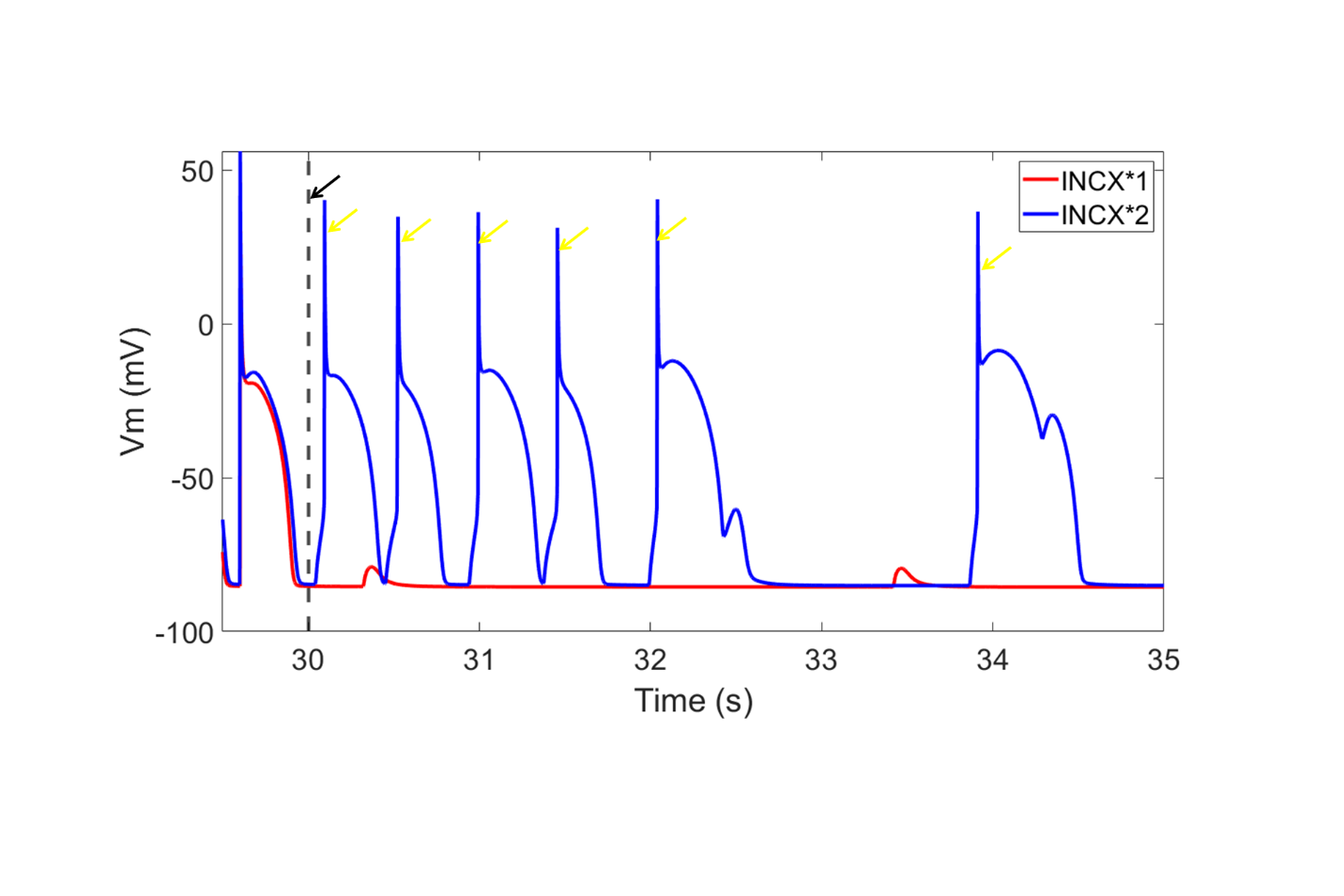


**Fig S4. Response of the rabbit Purkinje model to sodium-calcium exchanger current (INCX) increase after complete IKr blockade.**

1. Evolution of action potential with time when INCX in control model (red). **B**. Evolution of action potential with time when INCX is raised to 2 folds (blue trace with yellow arrow). Pacing the model for 30 s and removing the stimulus (black dash line) resulted in triggered activity when INCX density was raised to 2 folds.


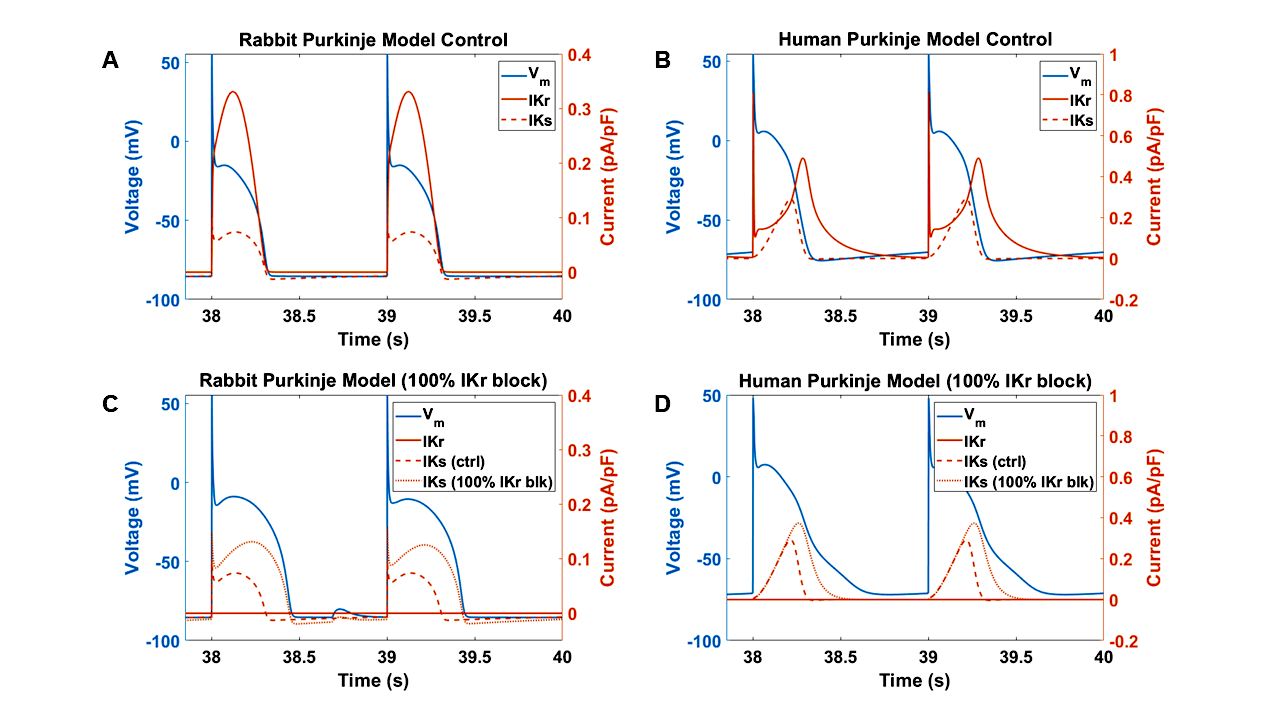


**Fig S5. Evolution of Action potential (AP) and repolarizing currents (IKr and IKs) with time at 1Hz pacing.**

1. Rabbit Purkinje AP model. **B**. Human AP model. **C**. Rabbit AP with 100% IKr block. **D**. Human AP with 100% IKr block. With IKr block entirely, the peak density of IKs current increases to compensate for the loss of IKr current. Ctrl- control, blk - block
